# The outcomes of endoscopic orbital decompression combined with fat decompression for thyroid-associated ophthalmopathy

**DOI:** 10.1186/s12886-023-02957-7

**Published:** 2023-05-16

**Authors:** Yunyan Ye, Feng Hu, Yuanfei Ji, Ruijun Wang, Kexuan Zhu, Qiao Kong

**Affiliations:** Department of Ophthalmology, Li Huili Hospital affiliated with Ningbo University, Xingning Road-57, Yinzhou District, Ningbo City, Zhejiang Province China

**Keywords:** Graves, Exophthalmos, Elevated IOP, Decompression

## Abstract

**Purpose:**

To present the clinical features of thyroid-associated ophthalmopathy (TAO) with different CT types, and to report the outcomes of endoscopic orbital decompression combined with fat decompression (EOD-FD).

**Patients and methods:**

Thirty-four patients with TAO who underwent EOD-FD between December 2020 and March 2022 in the Ophthalmology Department of Li Huili Hospital Affiliated with Ningbo University, were included in this retrospective interventional case series. Patients were categorized into two groups based on the results of computerized tomography (CT) scans: muscle expansion type and fat hyperplasia type.

**Results:**

Thirty-four TAO patients (55 eyes) were included in this study, and the mean age was 38.62 years (range 22–60 years). The average eye protrusion (EP) reduced from preoperative 23.20 mm to postoperative 19.66 mm (*p* < 0.0001). Mean intraocular pressure (IOP) decreased from 20.11 mmHg at baseline to 17.29 mmHg postoperatively (*p* < 0.0001), with a reduction of 2.84 mmHg (14.12%). Twenty cases of muscle expansion and fourteen cases of fat hyperplasia were definite by CT imaging. The mean IOP in the muscle expansion group was higher than that in the fat hyperplasia group (*p* < 0.05). Elevated intraocular pressure (IOP) occurred in 23 eyes (36.11%), and it was associated with extraocular muscle involvement, gender, and EP. In 3 cases of impaired vision, the mean best corrected visual acuity (VA) improved from 0.4 preoperatively to 0.84 postoperatively (*p* < 0.01). There were 8 cases with visual field (VF) damage and/or corneal epithelium damage, and all these damages were reversible.

**Conclusion:**

In this study, we describe the clinical features and experience of EOD-FD in patients with TAO. EOD-FD is an effective technique in reducing IOP and proptosis, with a low incidence of postoperative diplopia.

## Background

Thyroid-associated ophthalmopathy (TAO), also called Graves' orbitopathy (GO), is an autoimmune disorder of the retrobulbar tissue most commonly associated with Graves’ hyperthyroidism (Graves’ disease) [1, 2]. Patients may be hyperthyroidism, hypothyroidism or euthyroid [3, 4]. The clinical symptoms and signs include eyelid retraction, exophthalmos, ocular dyskinesia, diplopia, and strabismus. Dysthyroid optic neuropathy (DON) is the most common cause of visual loss in TAO [5, 6].

Definitive treatment in TAO often requires sufficiently enlarge the orbital space by means of orbital decompression to reduce proptosis, release the optic nerve compression, and promote the visual function recovery [7]. Recent years, a novel endoscopic trans-ethmoidal orbital decompression has increasingly gained popularity as an effective approach for managing TAO [8–10]. Intraoperative delicate handling and orbital fat excision remains a challenge to orbital surgeons [11, 12].

In this study, we reported clinical and surgical outcomes of endoscopic orbital medial wall decompression combined with fat decompression (EOD-FD) in TAO, described our experience of extraconal fat and intraconal fat excision.

## Methods

### Patients and study design

All patients who underwent endoscopic orbital medial wall decompression combined with fat decompression (EOD-FD) with TAO at the Ophthalmology Department of Li Huili Hospital Affiliated with Ningbo University's between December 2020 to March 2022, were included in this retrospective study. Demographic profiles, thyroid function, activity and severity of TAO, laboratory testing, and orbital examinations were all recorded. Pre-operative and post-operative orbital examinations included the best corrected visual acuity (VA), intraocular pressure (IOP), eyeball proptosis (EP), slit lamp microscopy, fundus examination, visual field (VF), eyelid height (distance from upper and lower eyelid margins to corneal reflection spot), and ocular motility. All patients were evaluated orbits and paranasal sinuses with orbital computerized tomography (CT). All patients had inactive thyroid eye disease for at least 6 months prior to surgery, as defined by EUGOGO classification. All operations were carried out by a single experienced orbital surgeon (Dr. Qiao Kong), and all patients were followed up at least 3 months following surgery. This retrospective case series study was approved by the Ethical Committee of Li Huili Hospital affiliated with Ningbo University (No.KY2021PJ209) and adhered to the tenets of the Declaration of Helsinki.

### Diagnostic criteria

1. The diagnostic criteria for TAO are present if eyelid retraction occurs in association with objective evidence of thyroid dysfunction or abnormal regulation, exophthalmos, optic nerve dysfunction, or extraocular muscle involvement. The ophthalmic signs may be either unilateral or bilateral, and confounding causes must be excluded.
2. If eyelid retraction is absent, then TAO may be diagnosed only if exophthalmos, optic nerve dysfunction, or extraocular muscle involvement is associated with thyroid dysfunction or abnormal regulation, and no other cause for the ophthalmic features is apparent [13].

### Activity and severity

The clinical activity score (CAS) and disease severity assessment are used to evaluate TAO by EUGOGO classification. CAS consists of seven items, each worth one point: spontaneous retrobulbar pain, pain on attempted upward or downward gaze, redness of eyelids, redness of conjunctiva, swelling of caruncle or plica, swelling of eyelids, swelling of conjunctiva (chemosis). TAO is considered to be active if CAS is ≥ 3/7. A ten-item CAS, including an increase in exophthalmos ≥ 2 mm, a decrease of eye movements in any direction of gaze ≥ 8°, and a decrease of visual acuity ≥ 1 line on the Snellen chart during a period of 1–3 months, is useful to evaluate recent progression and the activity of TAO [14].

The severity of TAO assessed by EUGOGO classification.

1. Mild TAO

Patients whose features of TAO have only a minor impact on daily life insufficient to justify immunosuppressive or surgical treatment. They usually have one or more of the following: minor lid retraction (< 2 mm), mild soft-tissue involvement, exophthalmos < 3 mm above normal for race and gender, no or intermittent diplopia and corneal exposure responsive to lubricants.

2) Moderate-to-severe TAO

Patients without sight-threatening TAO whose eye disease has sufficient impact on daily life to justify the risks of immunosuppression (if active) or surgical intervention (if inactive). They usually have two or more of the following: lid retraction ≥ 2 mm, moderate or severe soft-tissue involvement, or exophthalmos ≥ 3 mm above normal for race and gender, inconstant or constant diplopia.

3) Sight-threatening TAO (very severe TAO)

Patients with DON and/or corneal breakdown [14, 15].

### Indications for surgery

1. Exophthalmos affected with moderate-to-severe and inactive TAO. Patients have a strong desire to improve their appearance.
2. Patients with DON and/or corneal breakdown treated with high-dose iv methylprednisolone (single doses of 500 to 1000mg) for three consecutive days, when re-evaluate a week later showed no response and/or deterioration of ophthalmic signs, urgent orbital decompression surgery is mandatory [14].

### Exclusion criteria

The study did not include patients who met one of the following requirements: (1) Patients in poor health condition who cannot tolerate surgery, such as those with coagulopathy, heart, liver, or lung disease. (2) Ineffective management of hyperthyroidism or active TAO symptoms. (3) Nasal lesions should be treated initially if there are nasal polyps, significant sinusitis, and other disorders. (4) A history of strabismus, primary glaucoma, other eye diseases, or orbital surgeries.

### Measurement methods

All patients underwent pre-operative and post-operative orbital examinations. IOP measured by an Icare tonometer, and all measurements were performed between 8:00 and 12:00 to minimize the diurnal fluctuation of IOP (24 h system). IOP was measured at the original eye position in patients with strabismus, whereas IOP was assessed at the primary eye position in patients without strabismus. Elevated IOP was defined as IOP beyond 21 mmHg. In this study, none of the patients had a history of elevated IOP or glaucoma. The preoperative examination also found no abnormal of anterior chamber angle.

**Fig. 1:**
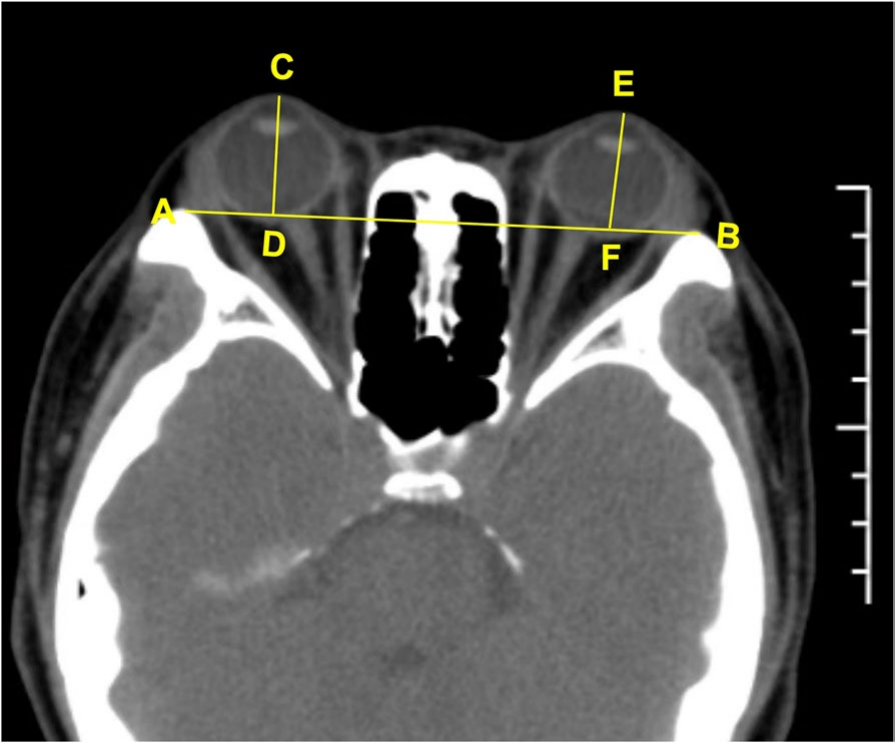
The measure exophthalmos based on CT imaging. AB is the orbital outer edge connection line, CD is the vertical distance from the corneal apex to the connection line of the right eye, which is the ocular protrusion. EF is the ocular protrusion of the left eye

EP measurements were performed by 2 methods. One method used Hertel exophthalmometry. The patient was seated facing the physician and instructed to face forward, and the notch of the Hertel exophthalmometry was embedded in the temporal orbital margin of the patient. It should be emphasized that when measuring EP for a patient, the orbital distance should be consistent before and after surgery. Another method measured EP based on the imaging of multi-slice spiral computerized tomography (MSCT) scan. The patient was supine on the scanning table with the head fixed and the eyeball fixed. The window width was 300–500 HU, the window level was 35–50 HU, and the layer thickness was 1 mm. All patients performed CT at primary eye position, and horizontal, coronal, and sagittal plane scanned at once. A window width of 400HU and a window position of 40HU were set for EP measurement. And the maximum diameter plane of the bilateral eyeball, the center of the lens, and the medial orbital segment of the optic nerve were all displayed in the same plane, and the outer orbital margin was at the lowest point. According to Fig. 1, AB denotes the connection line of the orbital outer edge, and CD denotes the vertical distance between the corneal apex and the connection line of the right eye, which is the eyeball proptosis. EF stands for left eyeball proptosis (Fig. 1).

Visual field examination used Humphrey computer automatic visual field analyzer (Germany Zeiss745i), with 30–2°, FAST mode, and white visual marker. Physiological blind spot is present a 6-to-8-degree vertical oval visual field defect, located 3 degrees below the horizontal position and 15 degrees from the center of fixation.

### Assessment and classification according to orbital CT imaging

**Fig. 2:**
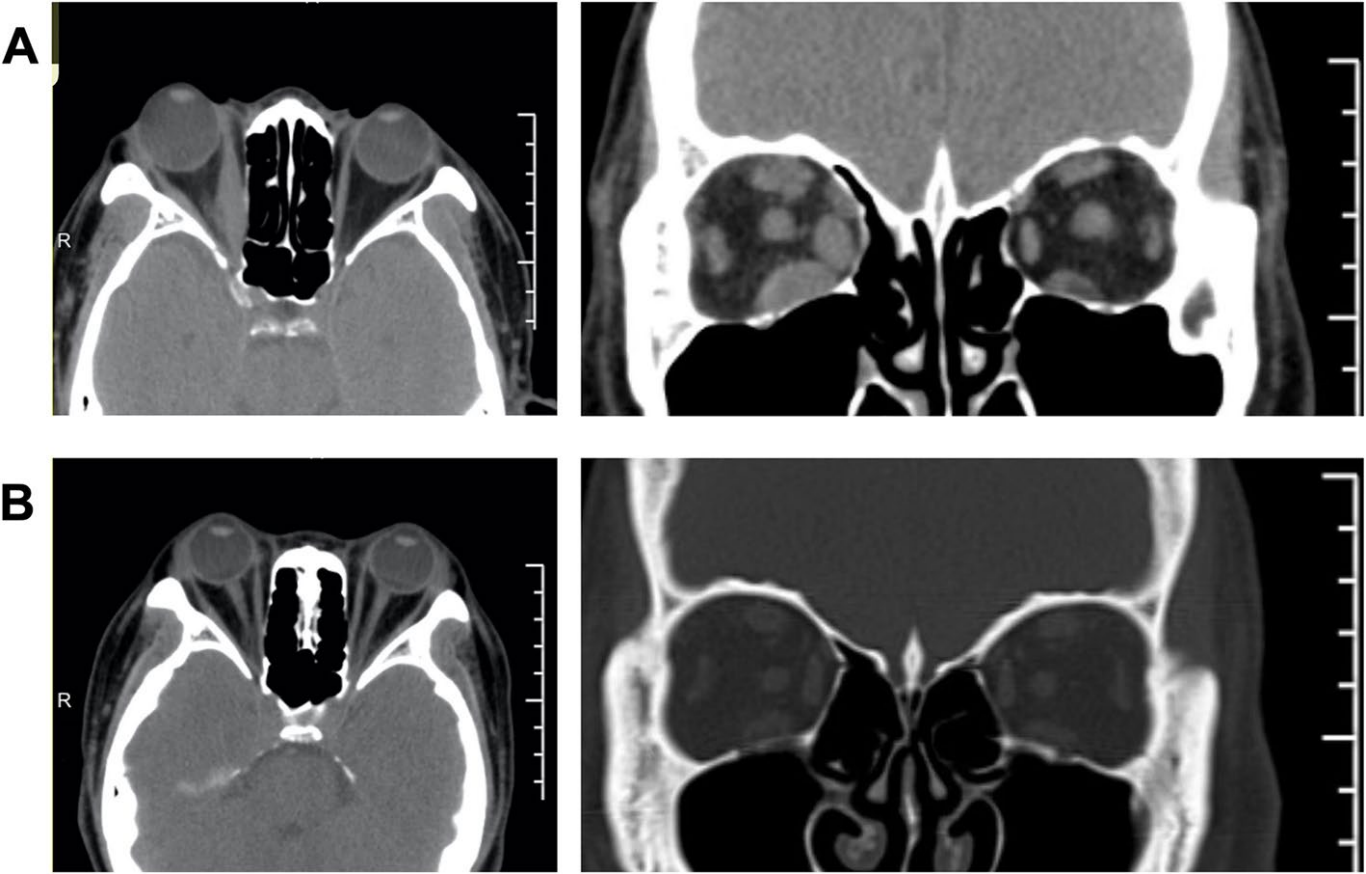
Imaging of TAO classification according to orbital CT scan. **A** Horizontal and coronal view of the muscle expansion type. CT scans revealed right eye protrusion, expansion of the inferior rectus, superior rectus, medial rectus muscles, and crowding of the orbital apex. **B** Horizontal and coronal view of the fat hyperplasia type. CT scans revealed binocular protrusion, increased orbital adipose tissue, and crowding of the orbital apex

All patients performed orbital CT pre-operatively to evaluate their orbits and paranasal sinuses. The images were archived and sent to Multimodality Workplace (MWP), and multiplanar reconstruction (MPR) was used to assess the orbital volume, extraocular muscle involvement, and optic nerve compression. Furthermore, the patients were classified into two groups according to CT imaging: muscle expansion type and fat hyperplasia type (Fig. 2 A and B). The orbital CT was reexamined to assess the morphology of soft tissue herniation and orbital cavity enlargement one-month after surgery.

### Surgical technique

All patients underwent EOD-FD under general anesthesia, and optic nerve decompression was performed simultaneously on patients with DON. After nasal cavity rinsing with diluted iodophor solution, nasal vasoconstriction and decongestion was achieved using cottonoid with adrenaline (1: 80,000) and lidocaine (2%) twice for 5 min. Where a septal deviation prevented sufficient access, a septoplasty was performed. The middle turbinate was fixed gently to reveal uncinate process and ethmoid bullae. Under endoscopy, the uncinate process and the lateral wall of the ethmoidal sinus were excised to widen the sinus's natural aperture, penetrate the sphenoid sinus, and completely expose the medial orbital wall to the orbital apex with a combination of a microcurette and Freer’s elevator. After gently removing the lamina papyracea to reveal the orbital apex, the skeletonized medial orbital wall was breached. A microdrill with a coarse diamond burr or an ultrasonic aspirator may be used to thin the bone down in the middle when it is too thick to be curetted down. The exposure range was posterior boundary to the junction of sphenoid lesser wing and optic nerve canal, anterior boundary to the maxillary line plane, upper boundary to the junction of medial orbital wall and anterior skull base, and lower boundary to the corner of the junction of medial orbital wall. We horizontally incised the periosteum from the orbital apex to the maxillary line using a sharp 9# MVR knife to create a periosteal band parallel to the medial rectus muscle, which was manipulated to extrude orbital fat into the ethmoid sinus [16].

**Fig. 3:**
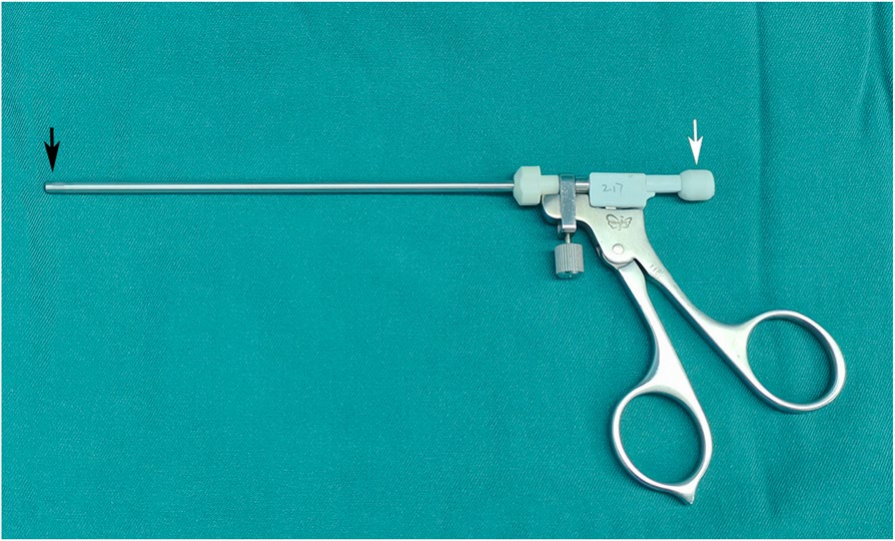
The unique suction/cutter instrument for endoscopic fat removal. The diagram identifies the cutting aperture (black arrow) and suction ports (white arrow)

Fat excision was performed using a special suction/cutter instrument (the New Direction Medical Optic Instrument Co, Ltd, Dezhou, P.R. China) (Fig. 3). This instrument consists of a negative pressure suction regulator (40–55 mmHg) and a cutting aperture, which is operated manually by compressing the handles. Cut the fat extruded in the ethmoid sinus, the fat behind the medial rectus muscle and part fat of fat pad, to expose the lower margin of the medial rectus muscle. The medial rectus muscle was gently lifted using a curved sphenoid sinus probe to expose intraconal fat. The titrated fat excision enabled further prolapse of intraconal fat into the sinus. Fat was carefully engaged into the suction cutter and cut under direct visualization of the nasal endoscopy in short bursts. If necessary, according to the correction needs of the eyeball protrusion, the assistant presses the finger above and below the outer orbit to promote prolapse of intraconal fat. During the operation, if the orbital blood vessels or important tissues are found to be sucked, the pressure handle should be pushed forward immediately to relieve the negative pressure immediately, to prevent vascular and extraocular muscle injury. Once the endoscopic fat decompression was completed, the longitudinal orbital fascia strip should be reduced to cover the surface of the medial rectus muscle. The protrusion and eye position should be cared during the procedure [17].

It was important to adequate hemostasis after surgery, with an absorbable gelatin sponge stuffed into the nasal cavity. The patient recovered from anesthesia and returned to the ward [18].

### Post-operative management

Intravenous methylprednisone 500mg Qd for 3 days, broad-spectrum antibiotics for 3 days to prevent infection [19]. Budesonide nasal spray was sprayed on the side of the nasal cavity twice daily in a bid to avoid adhesions and reduce postoperative inflammatory reactions. Post-operatively patients were advised to avoid blowing their nose and strenuous physical activity for 2 weeks. They were reviewed at 2-week and1,2,3 months after surgery, and postoperative VA, IOP, diplopia, EP, and eye movements were recorded at each review. Two weeks after the operation, nasal endoscopy was performed to remove the secretions in the ethmoid cavity and observe the wound healing. Orbital CT was reexamined 1 month after surgery. Patients with diplopia and eyelid retraction usually undergo corrective strabismus and/or eyelid surgery in the third month after EOD-FD, and then reviewed 3–6 months as required.

### Statistical analysis

Statistical analysis was performed using Prism 9 software. Measurement data with normal distribution are represented by mean while measurement data without normal distribution are represented by median and percentile. For comparison of measurement data, paired t-test was used for data distribution consistent with normal distribution, and the signed rank sum test was used for data distribution inconsistent with normal distribution. p < 0.05 was considered statistically significant.

## Results

### Clinical data at the initial visit

**Fig. 4:**
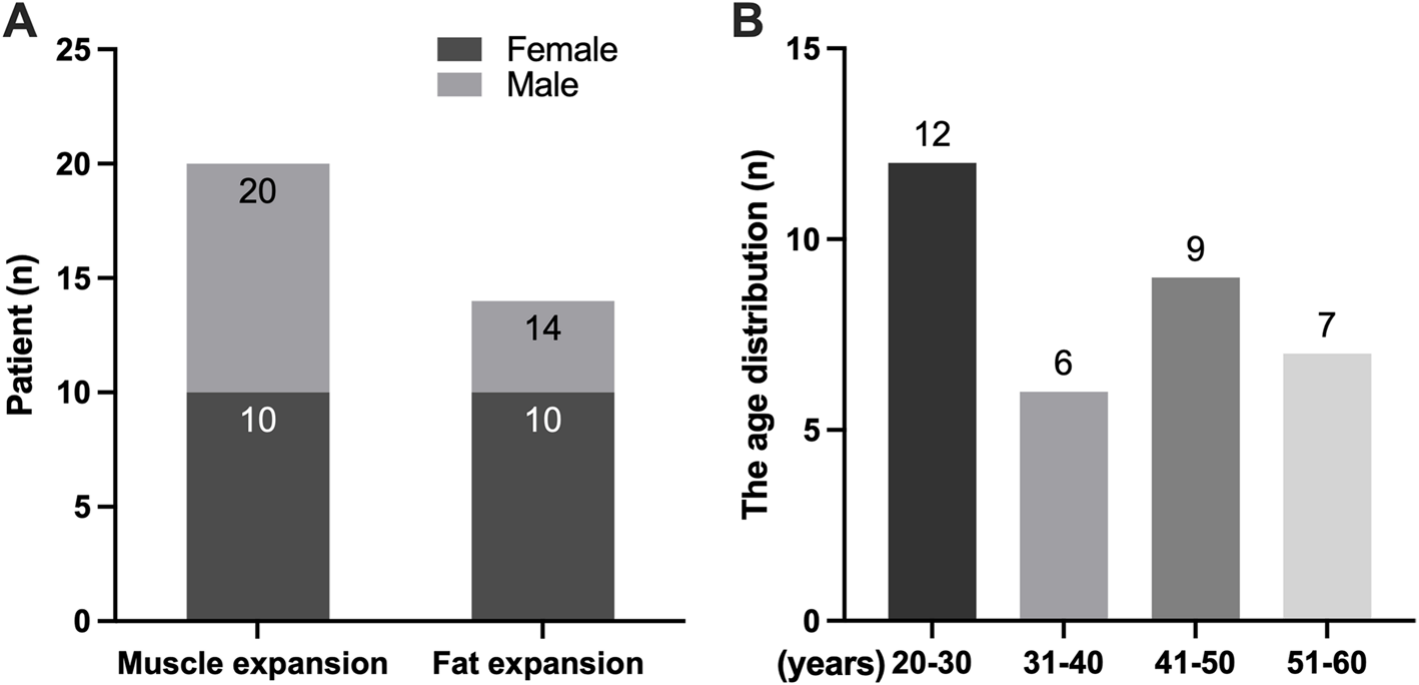
Gender and age distribution. **A** The gender distribution of the muscle expansion type and fat hyperplasia type. As the graph shows that there were 20 cases of muscle expansion type, and the male-to-female ratio was 1:1. There were 14 cases of fat hyperplasia type, and the male-to-female ratio was 1:2.5. **B** The age distribution of all cases. As the graph shows that the age distribution was 20–30 years old in 12 cases, 31–40 years old in 6 cases, 41–50 years old in 9 cases, and 51–60 years old in 7 cases

In this study, 55 eyes from 34 TAO patients were included, and 21 cases (64.7%) were binocular. All patients had surgery whose demographics are summarized in Table 1. According to CT imaging results, there were 20 cases of muscle expansion type, with an average age of 45.05 years (range, 28–60 years), and the male-to-female ratio was 1:1. There were 14 cases of fat hyperplasia type, with an average age of 29.43 years (range, 20–44 years), and the male-to-female ratio was 1:2.5 (Fig. 4 A). The age distribution was 20–30 years old in 12 cases, 31–40 years old in 6 cases, 41–50 years old in 9 cases, and 51–60 years old in 7 cases (Fig. 4 B). According to the EUGOGO consensus, intravenous corticosteroid pulses were administered to patients with active TAO to achieve inactivity, and none of them had received treatment with I^131^. Patients were euthyroid for 6 months prior to surgery.

**Table 1:** Demographic profiles and clinical data

Patients	34
Eyes	55
Average age, years (range)	38.62 (20–60)
Right eye: left eye	29:26
Classified by CT	
Fat hyperplasia, n (eye)	14 (23)
Muscle expansion, n (eye)	20 (32)
Female: male, n	20:14
Muscle expansion group	10:10
Fat hyperplasia group	10:04
Proptosis reduction, mm (range)	3.44 (2.0–6.2)
Dysthyroid optic neuropathy, n (%)	11 (8.82%)
Vision loss, n	3
Visual field damage, n	8
Preoperative diplopia, n (%)	17 (50.0%)
Improved diplopia, n (%)	7 (41.18%)
Postoperative new-onset diplopia, n (%)	2 (5.88%)
History of Thyroid function	
Hyperthyroidism, n	23
Normal, n	11
TPOAb positive, n	9
TGAb positive, n	7
TRAb positive, n	14

*TPOAb* Thyroid peroxidase antibodies, *TGAb* Thyroglobulin antibodies, *TRAb* Thyrotrophin receptor antibody

### Clinical course

**Fig. 5:**
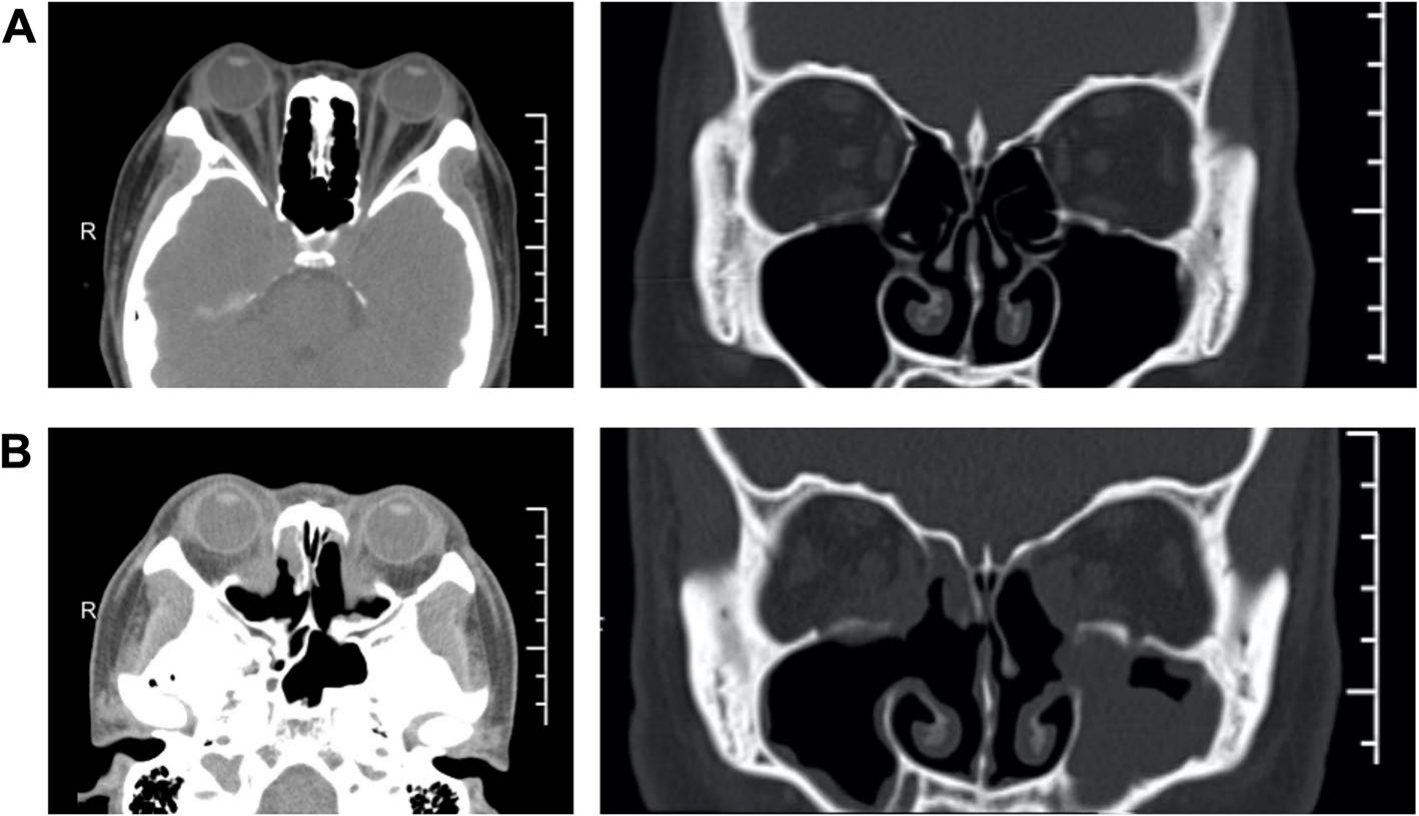
CT imaging before and after surgery. **A** Horizontal and coronal CT images before surgery. CT scans revealed binocular protrusion, increased orbital adipose tissue, and crowding of the orbital apex. **B** Horizontal and coronal CT images after surgery. A postoperative orbital CT revealed that the exophthalmos had significantly receded, most of the bone in the medial orbital wall's posterior part had vanished, the middle and posterior parts of the orbital cavity had enlarged, the fat herniation was uniform, and the extraocular muscle was not incarcerated

EOD-FD was performed on all patients, and simultaneous optic nerve decompression was performed on 11 patients (17 eyes). The postoperative proptosis was reduced obviously in comparison to the preoperative appearance. The eyelids were smooth with normal curvature, and there was no periorbital edema. A postoperative orbital CT revealed that the posterior part of medial orbital wall had removed, the middle and posterior parts of the orbital cavity had enlarged, the fat herniation was uniform, and the proptosis had receded significantly (Fig. 5). There was no complication such as cerebrospinal fluid leaking, frontal, and ethmoid sinus mucoceles, eye drops, eyelid droop, eye position alterations, sinusitis, or infraorbital nerve palsy. Patients receiving care for DON had no difference from those receiving care for aesthetic reasons. In 2 cases following surgery, newly developed diplopia spontaneously disappeared within one week. During the follow-up, 9 cases (14 eyes) underwent corrective strabismus surgery, and 11 cases (22 eyes) underwent eyelid surgery (Table 2). Parameters of strabismus surgery and eyelid surgery are summarized in Table 3.

**Table 2:** Surgical parameters

Endoscopic Orbital Decompression, n (eye)	34 (55)
Optic Nerve Decompression, n (eye)	11 (17)
Simultaneous bilateral surgery, n (eye)	10 (20)
Strabismus Surgery, n (eye)	9 (14)
Eyelid Surgery, n (eye)	11 (22)
Other’s complications, n	0

**Table 3:** Strabismus surgery and Eyelid surgery after decompression surgery, n (%)

**Strabismus surgery**, n (eye, %)	9 (14, 40.9%)
Medial rectus, n (eye)	4 (6)
Inferior rectus, n (eye)	2 (4)
Two extraocular muscles simultaneous surgery, n (eye)	3 (4)
Medial and lateral rectus muscles	1 (2)
Medial and inferior rectus muscles	2 (2)
**Eyelid surgery**, n (eye, %)	11 (22, 40.0%)
Eyelid retraction, n (eye, %)	10 (14,25.45%)
Lower eyelid entropion, n (eye, %)	7 (12, 21.81%)
Upper and lower eyelid simultaneous surgery, n (eye, %)	4 (8, 36.36%)
Secondary surgery, n (eye, %)	3 (3, 13.63%)

### The involvement of extraocular muscles

**Fig. 6:**
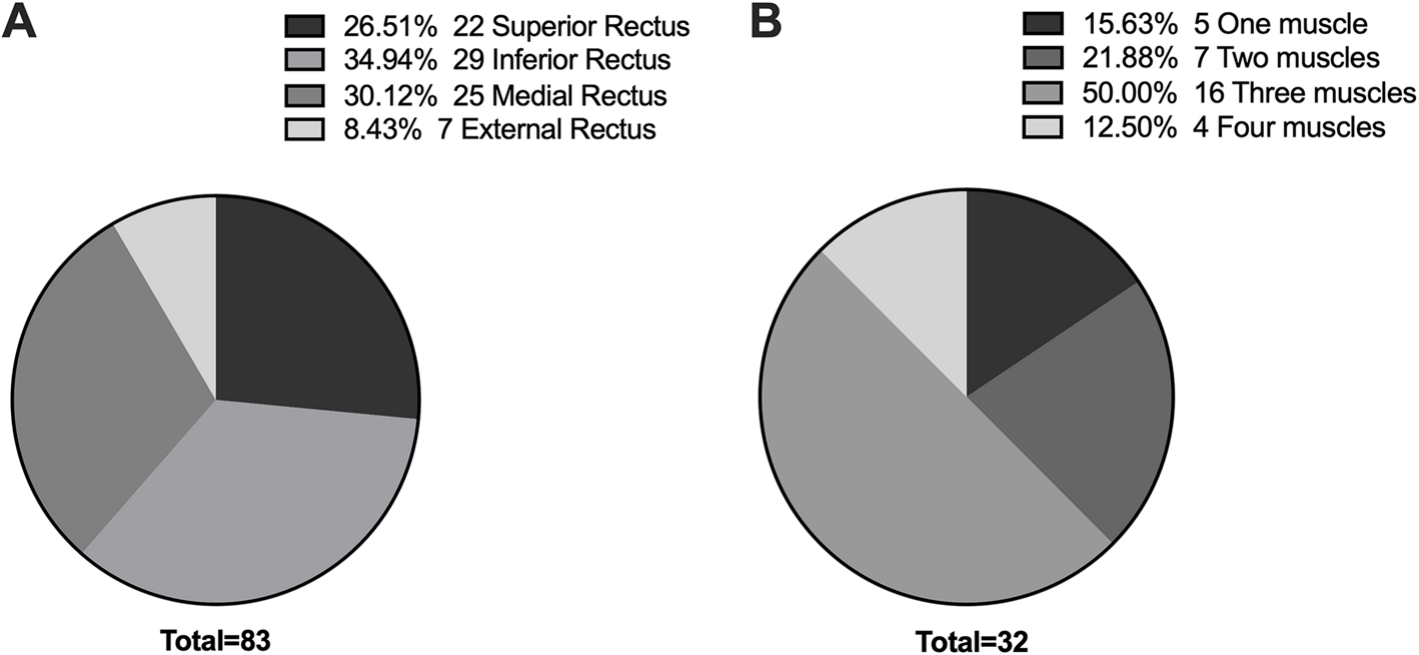
Involvement of extraocular muscles. **A** Frequency of each rectus muscle involvement. The expansion of the superior rectus muscle, inferior rectus muscle, medial rectus muscle, and lateral rectus muscle was 22 eyes (26.50%), 29 eyes (34.94%), 25 eyes (30.12%) and 7 eyes (8.43%), respectively.** B** The number of rectus muscles involved in each eye. One, two, three, and four extraocular muscle involvement was 5 eyes (15.63%), 7 eyes (21.88%), 16 eyes (50.00%), and 4 eyes (12.5%), respectively

Orbital CT showed extraocular muscle expansion in 32 eyes (58.18%) of 20 cases, mainly with muscle belly thickening. Monocular extraocular muscle was involved in 8 cases (40.0%), and binocular extraocular muscle was involved in 12 cases (60.0%). The involvement of extraocular muscles is summarized in Table 4. And frequency of each rectus muscle involvement and the number of rectus muscles involved in each eye are shown in Fig. 6.

**Table 4:** The involvement of extraocular muscles

**The frequency of involvement of each extraocular muscle**	
Superior rectus muscle, n (%)	22 (26.50%)
Inferior rectus muscle, n (%)	29 (34.94%)
Medial rectus muscle, n (%)	25 (30.12%)
Lateral rectus muscle, n (%)	7 (8.43%)
**Involvement of one rectus muscle, n**	**5**
Superior, n	2
Inferior, n	3
**Involvement of two rectus muscle, n**	**7**
Superior and Inferior, n	2
Superior and Medial, n	2
Inferior and Medial, n	2
Lateral and Medial,n	1
**Involvement of three rectus muscle, n**	**16**
Superior, Inferior and Medial,n	14
Inferior, Medial and Lateral,n	2
**Involvement of four rectus muscle, n**	4

### Relationship between extraocular muscle expansion and strabismus

Thirteen patients with strabismus were all muscle expansion type with diplopia, and most of them had multiple muscles involved in the binoculus. Ten of 13 patients with strabismus involved 3 muscles expansion. One of them had inferior, medial, and lateral rectus expansion with esotropia, and the other 9 patients had superior, inferior, and medial rectus expansion with esotropia and hypotropia. One of 13 patient with strabismus had superior and inferior rectus expansion with hypotropia. The other 2 of 13 patients had inferior and medial rectus expansion with esotropia and hypotropia. There was no significant change in eye position after the operation.

### Measurement of eyeball proptosis

**Fig. 7:**
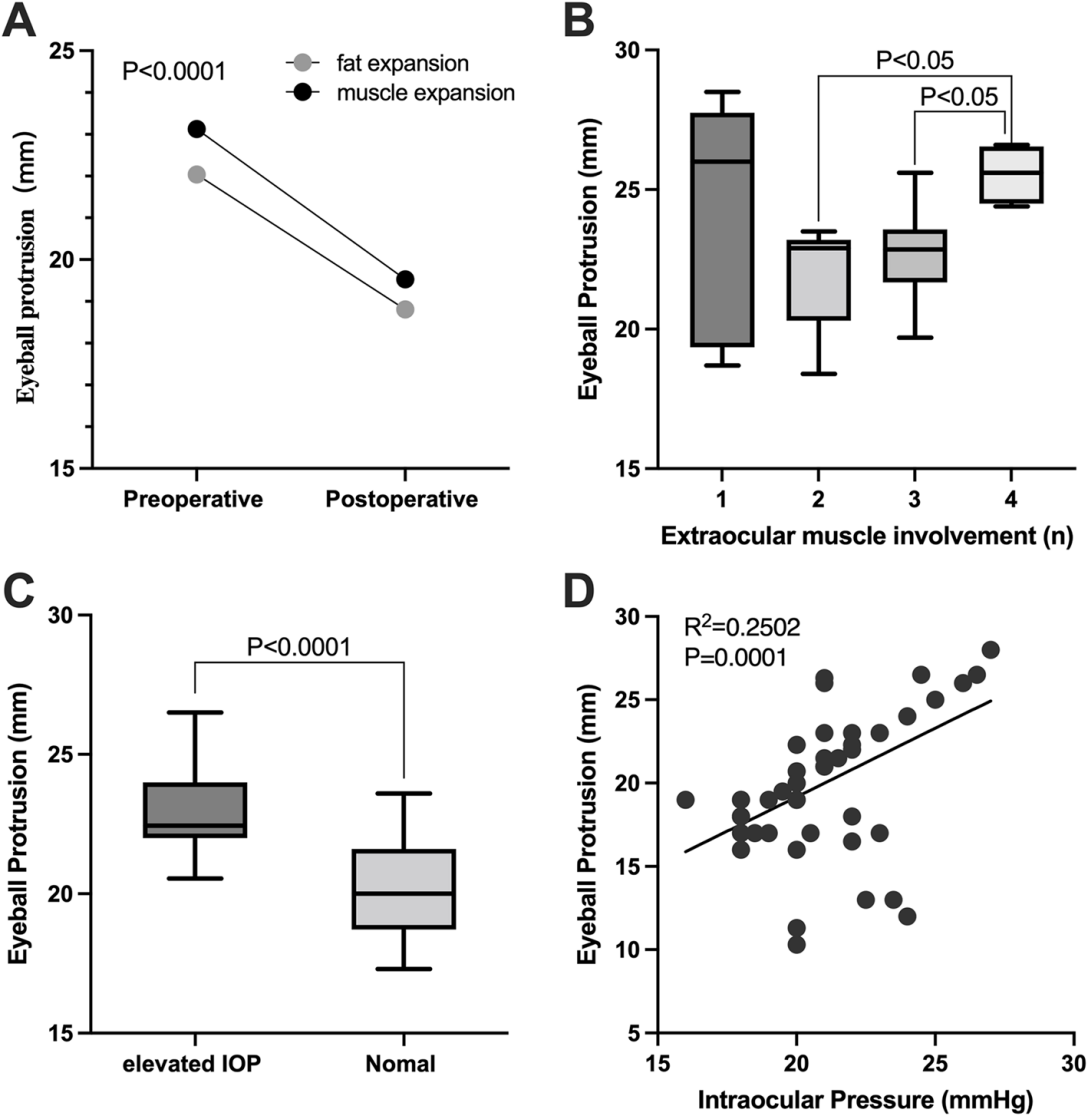
Eyeball protrusion and influencing factors. **A** The changes of eyeball protrusion in two types before and after surgery. **B** Relationship between extraocular muscle involvement and eyeball protrusion. **C** Eyeball protrusion in high IOP group and normal IOP group. **D** Linear correlation between eyeball protrusion and IOP

EP of CT measurement was higher than that of Hertel ophthalmostat meter significantly, with a mean difference of 1.93 mm (*p* < 0.0001). There was a linear correlation between the two methods (*p* < 0.001) (Table 5). EP was measured by CT imaging in all patients, and proptosis reduction was 3.44 mm. The preoperative and postoperative mean EP in each group are summarized in Table 5. There was a significant difference in EP between the two groups (*p* < 0.0001, Fig. 7 A). When l, 2, 3, and 4 extraocular muscles were involved, the EP was 24.04 mm, 22.03 mm, 22.73 mm, and 25.55 mm, respectively. When Bonferroni's method was used to adjust for a significance level, the distribution of IOP was statistically significant when the number of extraocular muscle involvement was 2 and 4 (adjusted *p* < 0.05), 3 and 4 (adjusted *p* < 0.05), but there was no significant difference in other groups (Fig. 7 B).

**Table 5:** Preoperative and postoperative clinical indicators

**Groups**		**Eye protrusion (mm)**				**IOP**
		CT	Hertel	PR	*P* value	**(mmHg)**
All	Pre	23.1	21.15	1.93	< 0.0001	20.11
	Post	19.66	17.72	1.94	< 0.0001	17.29
	PR	3.44	3.43	0.01	> 0.5	2.84
	*P* value	< 0.0001	< 0.0001	> 0.5		< 0.0001
Muscle expansion	Pre	23.13	21.19	1.94	< 0.0001	20.81
	Post	19.53	17.62	1.91	< 0.0001	17.83
	PR	3.58	3.57	0.01	> 0.5	3.02
	*P* value	< 0.0001	< 0.0001	> 0.5		< 0.0001
Fat hyperplasia	Pre	23.04	21.09	1.91	< 0.0001	19.14
	Post	19.81	17.89	1.92	< 0.0001	16.54
	PR	3.23	3.21	0.02	> 0.5	2.6
	*P* value	< 0.0001	< 0.0001	> 0.5		< 0.005

*IOP* Intraocular Pressure, *Pre* Preoperative, *Post* Postoperative

### Intraocular pressure and possible influencing factors

**Fig. 8:**
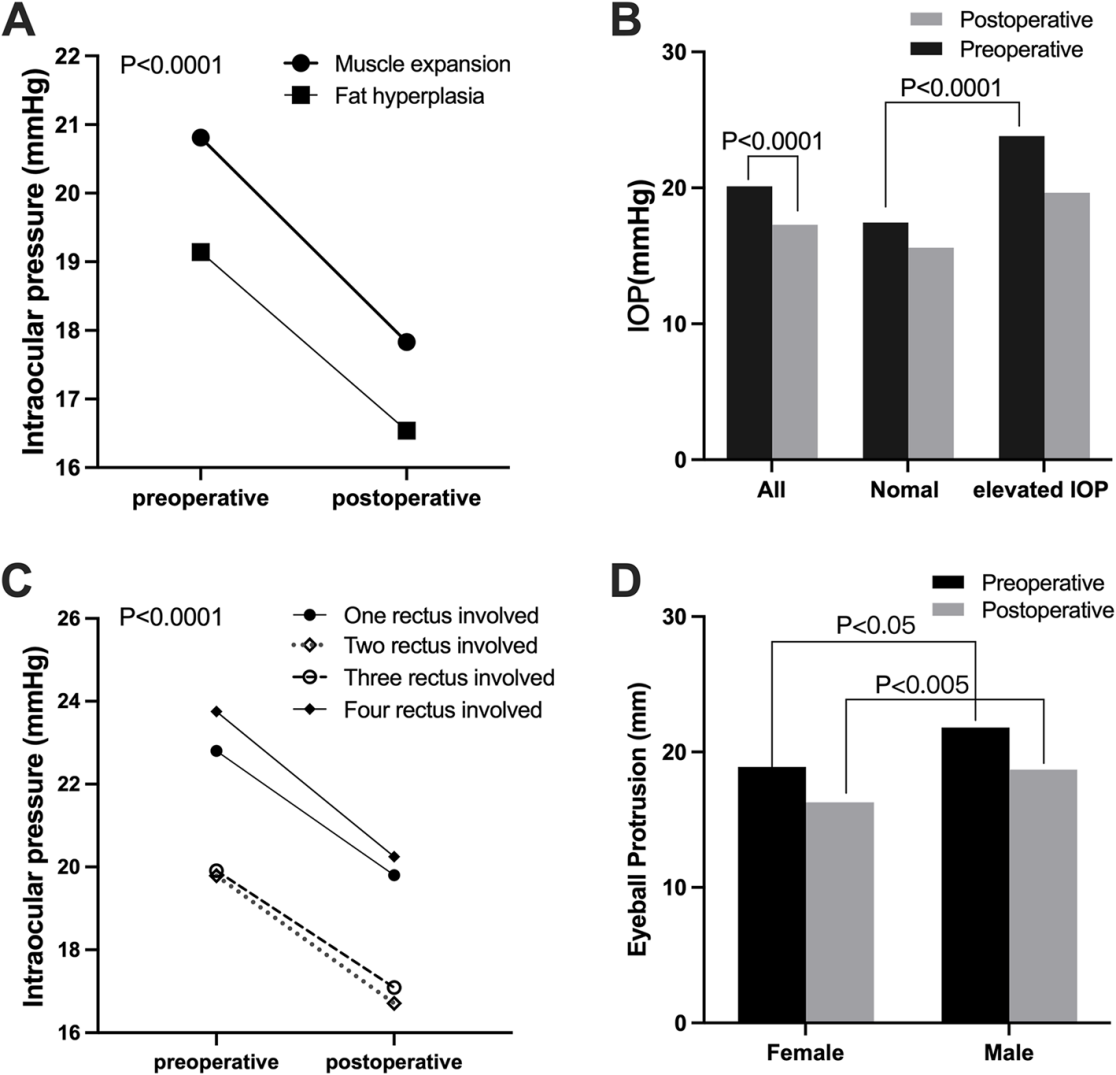
Intraocular pressure and influencing factors. **A** The changes of IOP in two types before and after surgery. **B** Changes of IOP before and after surgery in high IOP group and normal IOP group. **C** Relationship between extraocular muscle involvement and IOP. **D** Changes of IOP before and after surgery in females and males

In all cases, the mean IOP decreased from 20.11 mmHg at baseline to 17.29 mmHg postoperative, with an average reduction of 2.84 mmHg (*p* < 0.0001). In the muscle expansion group, the preoperative IOP was 20.81 mmHg and reduced to 17.83 mmHg after the operation, with a decrease of 3.02 mmHg (*p* < 0.0001). In the group with fat hyperplasia, the preoperative IOP was 19.14 mm and reduced to 16.54 mmHg after the operation, with a decrease of 2.60 mmHg (*p* < 0.0001). Both before and after the operation, the mean IOP in ocular muscle expansion group was higher than that in fat hyperplasia group (*p* < 0.05), but there was no significant difference in range of IOP decline between the two groups (Fig. 8 A B).

In all cases, the range of EP by CT imaging was 18.4–28.1 mm, and the incidence of elevated IOP was 41.82%. In the elevated IOP group, the average preoperative IOP was 23.82 mmHg (range 21.5–26.5), and the mean EP was 25.03 mm (range 22.7 mm-28.5 mm). In the normal IOP group, 32 eyes out of 21 cases had an average preoperative IOP of 17.45 mmHg (range 10.3–21) and mean EP of 21.71 mm (range 18.4 mm-25.6 mm), with a significant difference between the two groups (*p* < 0.0001, Fig. 8 B). According to Spearman correlation analysis, IOP and EP have a significant correlation (R^2^ = 0.2502, *p* < 0.0001, Fig. 7 C D).

In each group, according to involvement of extraocular muscle, the distribution of IOP was different (*p* < 0.05). Preoperative IOP in group of l, 2, 3, and 4 extraocular muscles involvement was 22.80 mmHg, 19.79 mmHg, 19.91 mmHg, and 23.75 mmHg respectively, and there was a statistical difference between the groups of 2 and 4 extraocular muscles involvement (adjusted *p* < 0.005), and the groups of 3 and 4 extraocular muscles involvement (adjusted *p* < 0.005). Postoperative IOP in group of l, 2, 3, and 4 extraocular muscles involvement was 19.80 mmHg, 16.71 mmHg, 17.09 mmHg, and 20.25 mmHg respectively. Postoperative IOP was lower than preoperative IOP in each group, and the difference was statistically significant (Fig. 8 C).

There were 20 females and 14 males in a total of 34 cases. Among the 20 female patients, 10 cases were muscle expansion type (50%), and 10 cases were fat hyperplasia type (50%). Among the 14 male patients, 10 patients were muscle expansion type (71.43%), and 4 patients were fat hyperplasia type (28.57%). The IOP in male patients was preoperative 21.80 mmHg and decreased to postoperative 18.7 mmHg, while IOP in female patients was 18.90 mm preoperative and decreased to 16.28 mm postoperative (*p* < 0.001). The average preoperative EP in male patients was 23.80 mm and decreased to postoperative 20.39 mm, while EP in female patients was preoperative 22.59 mm and decreased to postoperative 19.13 mm) (*p* < 0.001, Fig. 8 D). Both before and after surgery, EP and IOP in male patients was higher than that in female patients. There was no significant difference in the range of proptosis reduction between males and females (*p* > 0.5).

### Visual acuity and visual field

There were 11 cases of DON, 6 of them were bilateral, including 3 cases of VA impairment combined with visual field damage, and 8 cases of visual field damage and/or corneal epithelial damage. Optic nerve decompression surgery can significantly improve the visual impairment caused by compression optic neuropathy in TAO patients. The average AV of 3 patients with visual impairment increased from 0.4 before the operation to 0.8 after the operation. Visual field testing revealed significant visual acuity loss in all DON patients. In addition, two patients with fat hyperplasia of DON showed physiological blind spot enlargement and peripheral visual field narrowing. Among the 9 patients with muscle expansion type of DON, there were 3 cases of physiological blind spot enlargement, 2 cases of inferior arcuate dark spot, 2 cases of paracentric dark spot, and 2 cases of visual field constriction. One month after surgery, the visual field returned to normal in 90% of patients, and only mild abnormalities were left in 10% of patients. The mean defect value (MD) increased from -4.15 dB preoperatively to -0.85 dB postoperatively. Our study showed visual field defects unique to cases of compression optic neuropathy, including enlarged blind spots, and central and paracentral scotomas, all of which showed reversible changes.

## Discussion

Orbital decompression is an important surgical method for the treatment of exophthalmos in TAO patients, which is through destroying the orbit wall and increasing the orbital space to achieve proptosis reduction. [20] DON is the most common cause of visual loss in TAO. The most important purpose of orbital decompression is to relieved optic nerve compression and improved visual function in past. However, with the continuous improvement of living, medical and health conditions, correction of excessive exophthalmos and improvement of appearance has become another important purpose of orbital decompression. Especially for young patients with exophthalmos, there is an increasing demand for cosmetic surgery to improve their appearance [21]. Conventional orbital decompression through the anterior orbital approach (transcutaneous or conjunctival incision) has the disadvantages of more complications, inadequate proptosis reduction, and facial scar. In recent years, endoscopic orbital decompression has become one of the classic decompression methods. It can not only perform resection of the medial and inferior orbital wall, but also show the anatomical structure of the lacrimal duct and the optic nerve canal clearly [10, 22].

Autopsy studies have shown that the removal of the entire medial orbital wall can increase orbital volume by about 6ml [23], but clinical studies have found that proptosis reduction was 2.0–4.0 mm by endoscopic medial orbital wall decompression [24]. Michel et al. performed endoscopic medial orbital wall decompression in 145 eyes of 78 patients with TAO, and proptosis reduction was (3.94 ± 2.73) mm [25]. To increase the proptosis reduction, endoscopic medial wall decompression is often performed in combination with orbital inferior and lateral wall decompression. Schaefer et al. performed underwent endoscopic medial orbital wall decompression combined with transconjunctival orbital inferior decompression in 72 eyes and found that the proptosis reduction was 3.65 mm [26]. Jong Woo Kim et al. performed endoscopic medial wall decompression in 17 eyes out of 40 eyes, and the protrusion improved from 20.4 ± 1.16 mm before surgery to 16.8 ± 1.02 mm after surgery. The remaining 23 eyes underwent endoscopic medial wall decompression and transconjunctival inferior wall decompression, and the protrusion improved from 20.8 ± 1.75 mm before surgery to 14.8 ± 1.79 mm after surgery, but diplopia occurred in 2 cases after surgery [27]. Single orbital fat decompression was also limited in correcting exophthalmos, proptosis reduction ranging from 1.8–6 mm [28]. Therefore, endoscopic medial orbital wall decompression combined with fat decompression was adopted to achieve a better proptosis reduction, and the proptosis reduction was 4–9 mm in previous studies [29]. Zhang et al. performed endoscopic medial wall decompression combined with fat decompression in 29 patients with 45 eyes of TAO and found that proptosis reduction was 7.07 ± 1.59 mm, which was significantly higher than previously reported [8]. The average proptosis reduction was 3.44 mm in this study, which was lower than that of previous studies. The possible reasons were as follows: (1) Most of patients were aged 20–45 years (22 cases, 68.75%), who had a short course of TAO and had not developed into severe exophthalmos. They were more concerned about the effect of exophthalmos on their appearance, so they sought medical treatment in time and required surgical treatment to improve their appearance; (2) Some patients underwent surgical treatment for elevated IOP, visual field damage, and/or corneal damage rather than disfiguring exophthalmos; (3) Patients' awareness of the disease and health concerns have been continuously improved, and disfiguring exophthalmos has been rare.

DON is the most common cause of visual loss in TAO, which can lead to severe vision loss. The postulated pathogenesis of DON includes direct apical compression from the enlarged extraocular muscles and/or increased orbital fat, or indirectly arising from elevated pressure secondary to orbital edema and congestion [8, 21]. To adequately extend the medial wall decompression posteriorly to release excessive pressure on the optic nerve is essential for visual recovery [30]. TAO is common in women aged 40–60 years, with a male-to-female ratio of 1:1.6 to 1:2.4, and patients often develop DON 8 to 11 years after diagnosis of TAO [31]. Visual loss is the most common impairment, and other common impairments include ocular pain, abnormal color vision, abnormal visual range, etc. VEP visual function examination and visual field examination can detect visual impairment in TAO patients more sensitively at the early stage, and can judge the optic nerve injury to a certain extent [32]. Characteristic visual field defects include physiological blind spot enlargement, arcuate or vertical scoma, paracentric scoma, and constriction of the visual field, but it needs to be differentiated from glaucoma.

It has been reported that complications such as aggravated diplopia or new diplopia, sinusitis, infraorbital nerve palsy, eyeball subsidence, frontoethmoidal mucoceles, and cerebrospinal fluid leakage may occur after endoscopic orbital decompression. Therefore, the complications should be fully considered while reducing exophthalmos and orbital pressure in orbital decompression surgery. Compared with traditional endoscopic medial orbital wall decompression via ethmoidal approach, most of the bone at the junction corner of the orbital paper plate in front of the equator and the medial and inferior wall, as well as the longitudinal orbital fascia band were retained to overcome diplopia in this study. In case of restricted strabismus or aggravated strabismus after orbital decompression, corrective strabismus surgery can be performed 3 months after the operation, to relieve strabismus, diplopia, and eye movement disorders.

Extraocular muscle expansion is the most common pathological change in TAO, including inferior rectus, superior rectus, medial rectus, and lateral rectus. Superior rectus and inferior rectus involvement are associated with vertical strabismus and uncompensated diplopia. Previous studies have shown that the prevalence of elevated IOP in TAO patients ranged from 3.74% to 24%, which was higher than that in healthy controls, while the prevalence of glaucoma was not significantly different from that in healthy controls [33–35]. Elevated IOP in TAO patients is secondary elevated IOP, and the pathogenesis of elevated IOP has not been completely clarified. The possible mechanisms include inflammation, compression, and extraocular muscle expansion, increased orbital content, increased superficial scleral venous pressure, and glucocorticoids [36]. One study found that the IOP of TAO patients with inferior rectus muscle expansion was higher than that of normal controls, and the IOP of TAO patients who underwent inferior rectus recession was lower than that before surgery [37]. Another study found that the orbital fat hyperplasia of TAO patients was higher than that of normal controls by using magnetic resonance fat quantification technique [38]. Previous studies have found that there are differences in the distribution of IOP between a different number of extraocular muscles involved in one eye, and the IOP of patients with four rectus muscles involved is higher than that of patients without extraocular muscles involved. This study found that muscle expansion type is more likely to cause elevated IOP than the fat hyperplasia type, and 1 rectus muscle involvement and 4 rectus muscle involvement were closely related to increased IOP. Therefore, reducing the inflammatory reaction, proliferation, and fibrosis of extraocular muscles and orbital tissues, and increasing the volume of the orbit is beneficial to reduce the IOP in patients with TAO [37].

The shortcomings of this study are as follows:1) This study has the inherent shortcomings of retrospective study, and it needs to conduct a prospective randomized controlled study on the effects of EOD-FD on the improvement of protrusion, changes in extraocular muscle function, optic nerve recovery, and morbidity rate of diplopia. 2) The relationship between the changes of orbital volume and IOP before and after surgery needs further study; 3) Categorization of TAO according to CT scan divide the patients into three groups (fat, muscle, and compound), the compound was not included in our study. 4) This surgery requires quite sophisticated surgical skills and the pathophysiological basis of TAO, which should be performed with caution, and personalized design surgery according to the patient's condition, appearance, and expectation.

## Conclusion

In conclusion, clinical manifestations of TAO with different CT types have their own characteristics. In this study, the type of muscle expansion was more common in male, and it is more likely to result in elevated IOP. We believe that the EOD-FD is effective and safe in managing TAO requiring rehabilitative surgery. This procedure of combined extraconal fat and intraconal fat excision achieves the dual purpose of sufficient proptosis recovery and　IOP reduction and with a low incidence of surgically-induced diplopia.

## Data Availability

The datasets used and/or analyzed during the current study available from the corresponding author on reasonable request.

## Abbreviations

TAO: Thyroid-associated ophthalmopathy
GO: Graves' ophthalmopathy
EOD-FD: Endoscopic orbital decompression combined with fat decompression
CT: Computerized tomography
EP: Eye protrusion
IOP: Intraocular pressure
DON: Dysthyroid optic neuropathy
VA: Visual acuity
VF: Visual field
CAS: Clinical activity score
MSCT: Multi-slice spiral computerized tomography
MWP: Multimodality workplace

## References

[1] Brent GA (2008). Graves' disease. N Engl J Med.

[2] Tanda M, Piantanida E, Liparulo L, Veronesi G, Lai A, Sassi L, Pariani N, Gallo D, Azzolini C, Ferrario M (2013). Prevalence and natural history of Graves' orbitopathy in a large series of patients with newly diagnosed graves' hyperthyroidism seen at a single center. J Clin Endocrinol Metab.

[3] Bahn RS, Heufelder AE (1993). Pathogenesis of Graves' ophthalmopathy. N Engl J Med.

[4] Wang Y, Smith TJ (2014). Current concepts in the molecular pathogenesis of thyroid-associated ophthalmopathy. Invest Ophthalmol Vis Sci.

[5] Bahn RS (2010). Graves' Ophthalmopathy. N Engl J Med.

[6] Mallika P, Tan A, Aziz S, Alwi SS, Chong M, Vanitha R, Intan G (2009). Thyroid associated ophthalmopathy–a review. Malay Fam Physician.

[7] Woods RS, Pilson Q, Kharytaniuk N, Cassidy L, Khan R, Timon CV (2020). Outcomes of endoscopic orbital decompression for graves’ ophthalmopathy. Ir J Med Sci (1971-).

[8] Lv Z, Selva D, Yan W, Daniel P, Tu Y, Wu W (2016). Endoscopical orbital fat decompression with medial orbital wall decompression for dysthyroid optic neuropathy. Curr Eye Res.

[9] Maheshwari R, Weis E (2012). Thyroid associated orbitopathy. Indian J Ophthalmol.

[10] Mishra S, Maurya VK, Kumar S, Ankita, Kaur A, Saxena SK (2020). Clinical management and therapeutic strategies for the thyroid-associated ophthalmopathy: current and future perspectives. Curr Eye Res.

[11] Huang R, Li G, Wang K, Wang Z, Zeng F, Hu H, Jiang T (2021). Comprehensive analysis of the clinical and biological significances of endoplasmic reticulum stress in diffuse gliomas. Front Cell Dev Biol.

[12] Huang Y, Fang S, Zhang S, Zhou H (2020). Progress in the pathogenesis of thyroid-associated ophthalmopathy and new drug development. Taiwan J Ophthalmol.

[13] Bartley GB, Gorman CA (1995). Diagnostic criteria for Graves' ophthalmopathy. Am J Ophthalmol.

[14] Bartalena L, Baldeschi L, Boboridis K, Eckstein A, Kahaly GJ, Marcocci C, Perros P, Salvi M, Wiersinga WM (2016). The 2016 European Thyroid Association/European Group on Graves' Orbitopathy Guidelines for the Management of Graves' Orbitopathy. Eur Thyroid J.

[15] Bartalena L, Kahaly GJ, Baldeschi L, Dayan CM, Eckstein A, Marcocci C, Marino M, Vaidya B, Wiersinga WM (2021). The 2021 European Group on Graves’ orbitopathy (EUGOGO) clinical practice guidelines for the medical management of Graves’ orbitopathy. Eur J Endocrinol.

[16] Shah J, Ting J, Sindwani R (2021). Endoscopic orbital decompression: technical pearls and pitfalls of an evolving technique. Am J Rhinol Allergy.

[17] Curragh DS, Selva D (2019). Endoscopic orbital fat decompression for the management of proptosis in Grave’s orbitopathy using a laryngeal skimmer blade. Eye.

[18] Pletcher SD, Sindwani R, Metson R (2006). Endoscopic orbital and optic nerve decompression. Otolaryngol Clin North Am.

[19] Wu WC, Bo Y, Wang ML, Ling H, Wang QM (2011). [Endoscopic trans-ethmoid medial orbital wall decompression combined with intraconal fat decompression for Graves' ophthalmopathy]. Zhonghua Er Bi Yan Hou Tou Jing Wai Ke Za Zhi = Chin J Otorhinolaryngol Head Neck Surg.

[20] Jurek-Matusiak O, Brożek-Mądry E, Jastrzębska H, Krzeski A (2021). Orbital decompression for thyroid eye disease: surgical treatment outcomes in endocrinological assessment. Endokrynol Pol.

[21] Dolman P (2021). Dysthyroid optic neuropathy: evaluation and management. J Endocrinol Invest.

[22] Hatton MP, Rubin P (2005). Controversies in thyroid-related orbitopathy: radiation and decompression. Int Ophthalmol Clin.

[23] Stabile JR, Trokel SM (1983). Increase in orbital volume obtained by decompression in dried skulls. Am J Ophthalmol.

[24] Kingdom TT, Davies BW, Durairaj VD (2015). Orbital decompression for the management of thyroid eye disease: an analysis of outcomes and complications. Laryngoscope.

[25] Michel O, Oberlnder N, Neugebauer P, Neugebauer A, Rümann W (2001). Follow-up of transnasal orbital decompression in severe Graves’ ophthalmopathy.

[26] Schaefer SD, Soliemanzadeh P, Rocca D, Yoo GP, Maher EA, Milite JP, Rocca R (2003). Endoscopic and transconjunctival orbital decompression for thyroid-related orbital apex compression. Laryngoscope.

[27] Kim JW, Kang SM (2020). Surgical outcomes of endoscopic medial orbital wall decompression. J Craniofac Surg.

[28] Boboridis KG, Gogakos A, Krassas GE (2010). Orbital fat decompression for Graves' orbitopathy: a literature review. Pediatr Endocrinol Rev.

[29] Mourits MP, Bijl H, Altea M, Baldeschi L, Boboridis K, Currò N, Dickinson A, Eckstein A, Freidel M, Guastella C (2009). Outcome of orbital decompression for disfiguring proptosis in patients with Graves’ orbitopathy using various surgical procedures. Br J Ophthalmol.

[30] Neigel JM, Rootman J, Belkin RI, Nugent RA, Drance SM, Beattie CW, Spinelli JA (1988). Dysthyroid optic neuropathy: the crowded orbital apex syndrome. Ophthalmology.

[31] Saeed P, Rad ST, Bisschop PH (2018). Dysthyroid optic neuropathy. Ophthalmic Plast Reconstr Surg.

[32] Blandford AD, Zhang D, Chundury RV, Perry JD (2017). Dysthyroid optic neuropathy: update on pathogenesis, diagnosis, and management. Exp Rev Ophthalmol.

[33] Skalicky SE, Borovik AM, Masselos K, Pandya VB, Wang LW, Figueira EC, Wilcsek G, Francis IC (2008). Prevalence of open-angle glaucoma, glaucoma suspect, and ocular hypertension in thyroid-related immune orbitopathy. J Glaucoma.

[34] Da Silva F, de Lourdes Veronese Rodrigues M, Akaishi P, Cruz AAV (2009). Graves' orbitopathy: frequency of ocular hypertension and glaucoma. Eye.

[35] Behrouzi Z, Rabei HM, Azizi F, Daftarian N, Mehrabi Y, Ardeshiri M, Mohammadpour M (2007). Prevalence of open-angle glaucoma, glaucoma suspect, and ocular hypertension in thyroid-related immune orbitopathy. J Glaucoma.

[36] Betzler BK, Young SM, Sundar G (2022). Intraocular Pressure and Glaucoma in Thyroid Eye Disease. Ophthalmic Plast Reconstr Surg.

[37] Li X, Bai X, Liu Z, Cheng M, Li J, Tan N, Yuan H (2021). The effect of inferior rectus muscle thickening on intraocular pressure in thyroid-associated ophthalmopathy. J Ophthalmol.

[38] Kvetny J, Puhakka KB, Røhl L (2006). Magnetic resonance imaging determination of extraocular eye muscle volume in patients with thyroid-associated ophthalmopathy and proptosis. Acta Ophthalmol Scand.

